# Use of an unmanned aerial vehicle to support situation assessment and decision-making in search and rescue operations in the mountains

**DOI:** 10.1186/1757-7241-22-S1-P16

**Published:** 2014-07-07

**Authors:** Håkon B Abrahamsen

**Affiliations:** 1 Department of Research and Development, The Norwegian Air Ambulance Foundation, Drøbak, Norway; 2 Department of Global Health and Primary Care, University of Bergen, Bergen, Norway; 3 National Centre for Emergency Primary Health Care, Uni Research, Bergen, Norway

## Background

Every year 150 persons are killed by avalanches in North America and Europe. Search for victims is resource-intensive and time consuming to do if the avalanche spans a large geographical area in rough terrain. Survival decreases rapidly with time to extrication. More than half of avalanche victims are partial buried and visible on the surface.

The objective was to evaluate feasibility of using a rotor-wing unmanned aerial vehicle to support situation assessment in search and rescue operations in the mountains.

## Method

The study took place in the mountains of Setesdalen (Hovden) in Norway. Two simulated scenarios were set up: a) An avalanche (approximately 50x100 meters, 2 avalanche beacons buried in the snow) and b) an injured skier trapped in a narrow canyon with unstable snow overhead. We used a radio controlled multirotor UAV equipped with a three-antenna digital avalanche transceiver (Mammut Pulse Barryvox) for beacon search and a HD video camera (GoPro HD Hero 3) for visual air search. Live video, turn directions and digital signals from the avalanche beacon were wirelessly transmitted to a ground station. Beacon search in the avalanche was blinded to the pilot.

## Results

Remote aerial visual searches at different altitudes identified ski tracks into the canyon, objects in the snow and outer boundaries of the avalanche. Fine search from fixed altitude with an avalanche transceiver localized two avalanche beacons buried in the snow repeatedly.

## Conclusion

An UAV is an effective tool carrier and save time in rough terrain which spans a large geographical area. It is a safe supplement to human resources in high-risk work environments like an avalanche. An UAV equipped with an avalanche transceiver and a HD camera can enhance situation assessment and provide useful decision-making support for pre-hospital services and ski patrols in the mountains.
